# Supplementary material for: Status of coral reefs in Antigua & Barbuda: using data to inform management
Source: PeerJ. 2020 Jun 1;8:e9236. doi: 10.7717/peerj.9236 (PMC7271886; doi:10.7717/peerj.9236)
Supplement: Supplemental Information 1 [file peerj-08-9236-s001.docx]

| **Species considered Commercially Significant** | |
| --- | --- |
| Black Margate | Anisotremus surinamensis |
| Pluma Porgy | Calamus pennatula |
| Ocean Triggerfish | Canthidermis sufflamen |
| Jacks | Carangidae |
| Bar Jack | Caranx ruber |
| Coney | Cephalopholis fulva |
| Groupers | Epinephelidae |
| Rock Hind | Epinephelus adscensionis |
| Red Hind | Epinephelus guttatus |
| Red Grouper | Epinephelus morio |
| Nassau Grouper | Epinephelus striatus |
| Grunts | Haemulidae |
| Juvenile Grunt | Haemulon / Anisotremus |
| White Margate | Haemulon album |
| French Grunt | Haemulon flavolineatum |
| Snappers | Lutjanidae |
| Mutton Snapper | Lutjanus analis |
| Schoolmaster | Lutjanus apodus |
| Blackfin Snapper | Lutjanus buccanella |
| Cubera Snapper | Lutjanus cyanopterus |
| Gray Snapper | Lutjanus griseus |
| Dog Snapper | Lutjanus jocu |
| Mahogany Snapper | Lutjanus mahogoni |
| Lane Snapper | Lutjanus synagris |
| Black Grouper | Mycteroperca bonaci |
| Yellowmouth Grouper | Mycteroperca interstitialis |
| Gag | Mycteroperca microlepis |
| Scamp | Mycteroperca phenax |
| Tiger Grouper | Mycteroperca tigris |
| Yellowfin Grouper | Mycteroperca venenosa |
| Yellowtail Snapper | Ocyurus chrysurus |
| Permit | Trachinotus falcatus |
